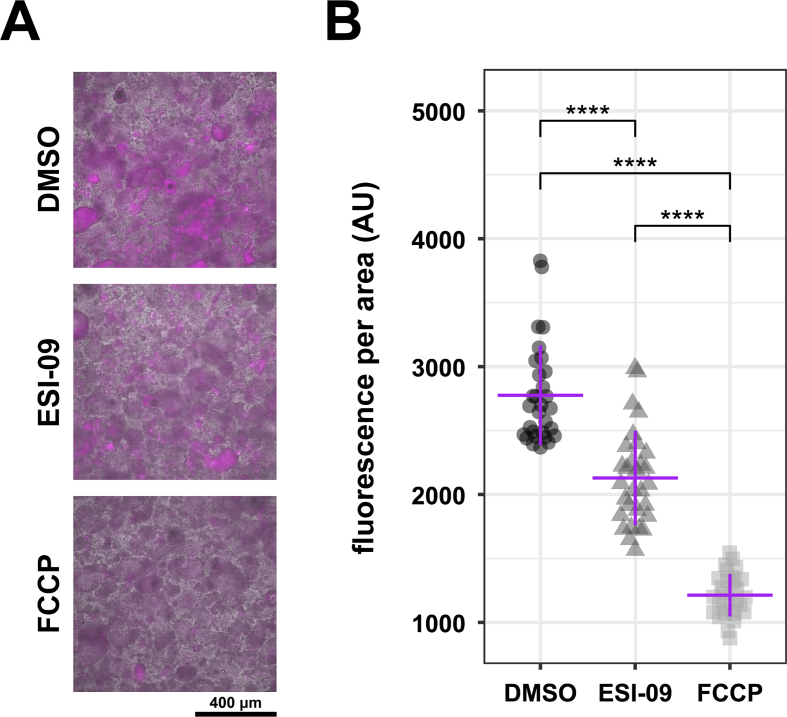# *In vitro* screening of the open-access Pandemic Response Box reveals ESI-09 as a compound with activity against *Echinococcus multilocularis*

**DOI:** 10.1016/j.ijpddr.2025.100609

**Published:** 2025-08-23

**Authors:** Pascal Zumstein, Anissa Bartetzko, Marc Kaethner, Laura Vetter, Andrew Hemphill, Trix Zumkehr, Benoît Laleu, Matías Preza, Britta Lundström-Stadelmann

**Affiliations:** a Institute of Parasitology, Vetsuisse Faculty, University of Bern, Bern, Switzerland; b Graduate School for Cellular and Biomedical Sciences, University of Bern, Bern, Switzerland; c MMV Medicines for Malaria Venture, ICC, Route de Pré-Bois 20, 1215, Geneva, Switzerland; d Multidisciplinary Center for Infectious Diseases, University of Bern, Bern, Switzerland

**Keywords:** *Echinococcus mutlilocularis*, Alveolar echinococcosis, Drug repurposing, MMV, DNDi, Mitochondrial uncoupler

## Abstract

Alveolar echinococcosis (AE) is a life-threatening disease caused by the metacestode stage of the fox tapeworm *Echinococcus multilocularis*, primarily in the liver. Current drug treatments rely on benzimidazoles, which are not parasiticidal, requiring life-long therapy with significant side effects. Therefore, novel drug treatments are urgently needed. Drug repurposing offers a strategy to identify novel therapies against the neglected disease AE. We report on the *in vitro* screening of the Pandemic Response Box, an open-access compound library composed of 400 drug-like molecules assembled by Medicines for Malaria Venture (MMV) and the Drugs for Neglected Disease Initiative (DNDi), against *E. multilocularis*. An overview screen at 10 μM using the metacestode vesicle damage-marker release assay (based on release of phosphoglucose isomerase, PGI) and metacestode vesicle viability assay (based on ATP measurement) identified 37 active compounds. Reassessment in triplicates resulted in five active compounds (alexidine, carbendazim, ESI-09, MMV1581545, oxfendazole) displaying anti-metacestode activity. The parasiticidal activity of these five compounds was evaluated by ATP measurement in germinal layer cells. One compound, ESI-09, acted specifically against *E. multilocularis* (IC_50_ on metacestode vesicles 6.06 ± 3.18 μM by PGI release assay and 2.09 ± 0.56 μM by metacestode vesicle viability assay as well as an IC_50_ of 2.45 ± 0.86 μM on germinal layer cells) with a broad therapeutic window when compared to mammalian cell toxicity. Further experiments applying Seahorse technology and tetramethylrhodamine ethyl ester (TMRE) assay revealed that ESI-09 acts as a mitochondrial uncoupler in parasite cells. However, transmission electron microscopy showed no significant ultrastructural changes in parasite mitochondria, though increased secretion of extracellular vesicle-like structures between the tegument and the laminated layer was observed. In summary, screening of the Pandemic Response Box identified ESI-09 as a potential drug candidate for the treatment of AE. Further experiments are needed to evaluate the efficacy of ESI-09 *in vivo*.

## Highlights

• The MMV Pandemic Response Box was screened agains*t E. multilocularis in vitro.*
• ESI-09 was the most potent out of 400 compounds against *E. multilocularis.*
• ESI-09 acts as a mitochondrial uncoupler in *E. multilocularis* germinal layer cells.

## Graphical abstract

**Figure:**
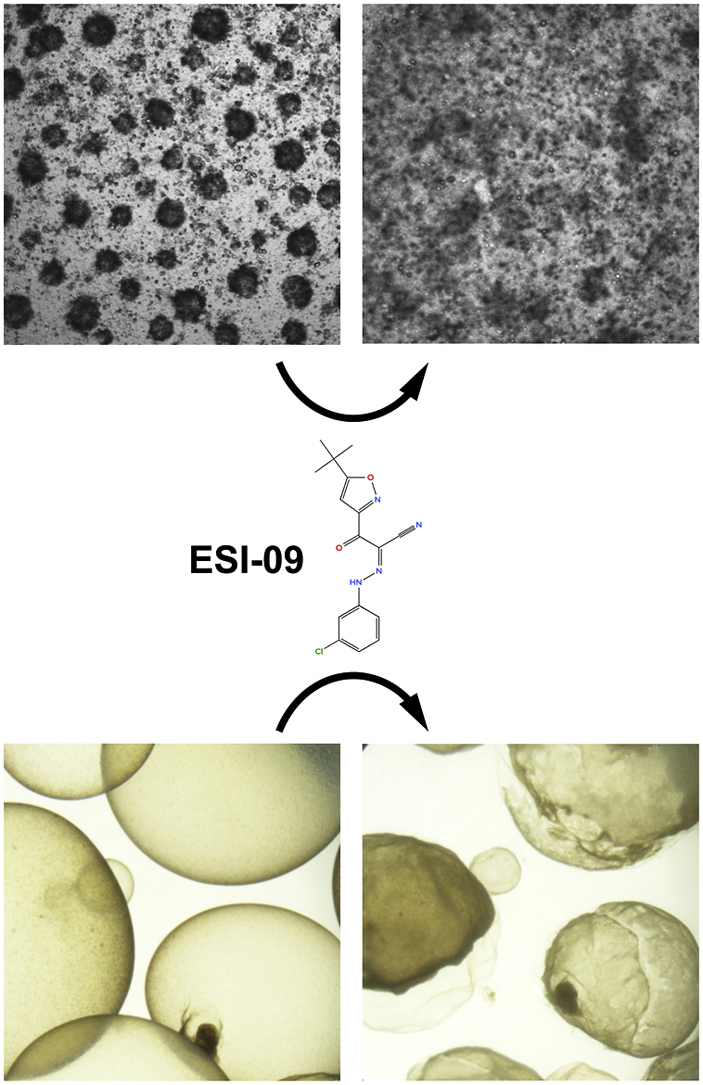

## 1 Introduction

The larval metacestode stage of the fox tapeworm *Echinococcus multilocularis* causes the severe zoonotic and neglected disease alveolar echinococcosis (AE) in humans and other mammals such as dogs and various species of simians (Corsini et al., 2015; Eckert and Deplazes, 2004). AE is acquired via ingestion of infectious eggs, from which oncospheres hatch. These move mostly to the liver, where they differentiate into the disease-causing metacestode (Eckert and Deplazes, 2004). AE is characterized by infiltrative, cancer-like growth of the parasite. In addition, metastases can form and spread to secondary organs (Eckert and Deplazes, 2004; Reinehr et al., 2020). If left untreated, AE is fatal. Annually, there are approximately 10′489 new AE cases (Lundström-Stadelmann et al., 2025), and the global impact was calculated to 688′000 disability adjusted life years (Torgerson et al., 2015). Treatment of choice is complete resection of the entire parasite mass by surgery, but this is only applicable in 20–50 % of cases in countries with well-developed health infrastructure (Grüner et al., 2017). Surgery is always accompanied by treatment with the benzimidazoles (BMZs) albendazole or mebendazole during extended periods of time (Brunetti et al., 2010). If curative surgery is not feasible, BMZ treatment is the only option. However, in most cases, BMZs act parasitostatically rather than parasiticidally, thus they stop parasite growth, but do not kill, and once treatment is interrupted, the parasite will regrow (Reuter et al., 2004). Therefore BMZs have to be administered daily and often life-long (Brunetti et al., 2010). Long-term administration of these drugs can lead to adverse effects including severe liver toxicity affecting up to 6.9 % of AE patients (Grüner et al., 2017). To prevent adverse effects, liver enzymes and drug serum levels have to be monitored regularly, which is highly dependent on infrastructure and is not always feasible (Brunetti et al., 2010). Still, clinical studies and long-term cohort data suggest that drug therapy has dramatically improved survival in inoperable AE patients, from a 10-year mortality rate of around 90 % prior to any drug treatment option to a median survival extending beyond 13 years (Deibel et al., 2025). Besides BMZs, the anti-fungal amphotericin B, the thiazolide nitazoxanide and the ant-malarial mefloquine have been repurposed for the treatment of AE and applied clinically: Amphotericin B was used in patients as a salvage treatment, but it was not parasiticidal, and upon long term usage induced nephrotoxicity (Tappe et al., 2009). Despite displaying promising activities in mouse studies, nitazoxanide and mefloquine failed in human AE treatment (Jelicic et al., 2023; Lundström-Stadelmann et al., 2020; Tappe et al., 2009).

Metacestode vesicles are *in vitro* generated subunits of the disease-causing metacestodes found in patients. They consist of an outer wall made of a carbohydrate-rich, acellular laminated layer, a cellular, syncytial tegument layer, and the cellular germinal layer (GL) (Koziol et al., 2014). Metacestode vesicles are filled with a fluid termed vesicle fluid. The GL consists of a variety of cells, of which around 20–30 % are undifferentiated stem cells that are responsible for the high regenerative potential of *E. multilocularis* metacestodes (Koziol et al., 2014). These stem cells are not affected by the current BMZs in use, at least in part explaining their parasitostatic effect (Brehm and Koziol, 2014; Schubert et al., 2014). Thus, for a compound to be useful for AE treatment, it must display activity on *E. multilocularis* stem cells. Advances in the development of *in vitro* culture techniques for metacestode vesicles and parasite cells, as well as the establishment of reliable, standardized and objective drug-screening assays, have greatly improved possibilities to identify novel active drugs (Lundström-Stadelmann et al., 2019; Spiliotis et al., 2004, 2010). A number of lead compounds with high efficacy against *E. multilocularis in vitro* have been identified, such as, endochin-like quinolones, SGI-1776 or CX-6258, to name a few (Chaudhry et al., 2022; Koike et al., 2022). However, neglected tropical diseases (NTDs) such as AE do not attract high financial investments and drug discovery research is mostly performed by academic laboratories with highly limited resources, hence drug repurposing offers a pragmatic and efficient approach to identify new treatments for NTDs (Klug et al., 2016). Open-source drug libraries are instrumental in early drug discovery and identification of new leads or novel drug scaffolds. This approach is supported by organizations such as the Medicines for Malaria Venture (MMV) (Wells et al., 2016). The first open-access drug library launched by MMV was the Malaria Box in 2013, a drug library consisting of 200 drug-like and 200 probe-like molecules with *in vitro* activity against *Plasmodium falciparum* (Spangenberg et al., 2013) and screening of this library led to the identification of the salicylanilide-derivative MMV665807 as a lead compound against AE (Stadelmann et al., 2016). MMV launched a successor Box, the Pathogen Box, in 2015, containing 400 diverse, drug-like molecules in different stages of clinical application. The Pathogen Box led to the discovery of the specific activity of the hydroxynaphthoquinone buparvaquone against the *E. multilocularis* (Rufener et al., 2018). However, both of these compounds failed to reduce parasite burden in the AE mouse model (Rufener et al., 2018; Stadelmann et al., 2016). Additionally, both the Malaria Box and the Pathogen Box were screened against various organisms, leading to the identification of several compounds with *in vitro* activities against unicellular parasites, including *Plasmodium falciparum* (Ahyong et al., 2016; Lucantoni et al., 2013; Mbye et al., 2023), *Toxoplasma gondii* (Boyom et al., 2014; Maus et al., 2024; Spalenka et al., 2017) or *Neospora caninum* (Müller et al., 2020), but also helminth parasites such as *Schistosoma mansoni* (Ingram-Sieber et al., 2014; Maccesi et al., 2019). Amongst the *in vitro* identified active MMV compounds, MMV007224 and MMV022478 reduced worm burden significantly in an *in vivo* mouse model of chronic *S. mansoni* infection (Ingram-Sieber et al., 2014; Pasche et al., 2019).

In this study, we screened the third generation of MMV libraries, the Pandemic Response Box, which was released in 2019 in collaboration with DNDi (Samby et al., 2022). We tested the 400 compounds (201 antibacterials, 153 antivirals and 46 antifungals) *in vitro* against *E. multilocularis* metacestode vesicles and active compounds against GL cells. Three promising compounds were further tested for their mammalian cell toxicity to assess for a potential therapeutic window. We identified one strongly active compound *in vitro*, for which we further analyzed the mode of action, as outlined in this study.

## 2 Materials and methods

### 2.1 Chemicals and reagents

If not stated otherwise, all chemicals were purchased from Sigma-Aldrich (Buchs, Switzerland) and all plastic ware was purchased from Sarstedt (Sevelen, Switzerland). Dulbeccos's modified Eagle medium (DMEM) and penicillin and streptomycin (10′000 U/mL penicillin, 10′000 μg/mL streptomycin) were purchased from Gibco (Fisher Scientific AG, Reinach, Switzerland). Fetal bovine serum (FBS) and Trypsin/EDTA (0.05 % Trypsin/0.02 % EDTA) were purchased from Bioswisstec (Schaffhausen, Switzerland). Reuber rat hepatoma (RH) cells (H-4-II-E) were purchased from ATCC (Molsheim Cedex, France). The Pandemic Response Box and compounds were provided by Medicines for Malaria Venture (Geneva, Switzerland) as part of the MMV Open program.

### 2.2 Mice and ethics statement

*E. multilocularis* strain H95 was maintained in female BALB/c mice (Charles River Laboratories, Sulzheim, Germany) as described previously (Kaethner et al., 2025). Mice were kept under controlled conditions with a 12-h light/dark cycle, a temperature of 21–23 °C and a relative humidity of 45–55 %. Food and water were provided *ad libitum*. Cages were enriched with nestlets (Plexx, Elst, Netherlands) and mouse houses (Tecniplast, Gams, Switzerland), tunnels (Zoonlab, Castrop-Rauxel, Germany). Experiments were approved by the Animal Welfare Committee of the canton of Bern under the license numbers BE30/19 and BE2/22 and the animals were treated in compliance with the Swiss Federal Protection of Animals Act (TSchV, SR455).

### 2.3 *In vitro* culture of *E. multilocularis* metacestode vesicles

*E. multilocularis* metacestode vesicles were cultured as described previously (Kaethner et al., 2023). Briefly, parasite material was excised from intraperitoneally infected BALB/c mice and pressed through a tea strainer (Migros, Bern, Switzerland). The material was incubated overnight at 4 °C in PBS containing penicillin (100 U/mL), streptomycin (100 μg/mL) and tetracycline (10 μg/mL). The next day, 1.5 mL of parasite material was co-cultured with RH cells in DMEM supplemented with 10 % FBS, penicillin (100 U/mL), streptomycin (100 μg/mL) and tetracycline (5 μg/mL) at 37 °C under a humid, 5 % CO_2_ atmosphere.

### 2.4 Assessment of drug efficacy against *E. multilocularis in vitro*

#### 2.4.1 Phosphoglucose isomerase (PGI) release assay on *E. multilocularis* metacestode vesicles

In order to quantify the drug-induced damage on whole metacestode vesicles, phosphoglucose isomerase (PGI) assay was performed as described previously (Stadelmann et al., 2010) with few adaptations published recently (Kaethner et al., 2023). In short, 8 to 12 weeks-old metacestode vesicles were purified using 2 % sucrose with subsequent washes in PBS. Metacestode vesicles were diluted in two volumes of DMEM without phenol red containing penicillin (100 U/mL) and streptomycin (100 μg/mL). 1 mL of the metacestode vesicle suspension was distributed to each well of a 48-well plate (Huberlab, Aesch, Switzerland) and the 400 compounds of the Pandemic Response Box were added to final concentrations of 10 μM in singlets for the overview screen and in triplicates in the confirmation screen (for active compounds of the overview screen, relative PGI activity ≥20 %, relative viability ≤30 %). 0.1 % DMSO was used as a negative solvent control and 0.1 % Triton X-100 was used as a positive total damage control. Metacestode vesicles were incubated under a humid, 5 % CO_2_ atmosphere at 37 °C. After five and twelve days, supernatant was taken and pictures were made using a Nikon SMZ18 stereo microscope (Nikon, Basel, Switzerland) at 7.5 × magnification. PGI activity in the supernatants was measured on a HIDEX Sense microplate reader (Hidex, Turku, Finland). The corresponding values of the DMSO controls were subtracted from the values of the compounds and then PGI activity was calculated relative to 0.1 % Triton X-100. Compounds inducing a relative PGI activity of 20 % were considered as active in the PGI release assay. IC_50_ calculations were carried out for ESI-09, testing this compound on metacestode vesicles at concentrations from 120 to 0.16 μM in 1:3 serial dilutions in triplicates. IC_50_ values were calculated applying the 3 parameter Weibull type 1 in R for each of the three independent experiments (Ritler et al., 2020).

#### 2.4.2 Assessment of *E. multilocularis* metacestode vesicle viability

Impact of compounds on the viability of entire metacestode vesicles was measured as described recently (Kaethner et al., 2023) with the difference that measurements were carried out in white 96-well plates. Viability was assessed after 12 days using the same metacestode vesicles as for the PGI release assay. Triton X-100 was added to each well to a final concentration of 0.1 %. Subsequently the metacestode vesicles were broken mechanically and 50 μL of the supernatant was mixed with 50 μL of CellTiter-Glo® buffer (Promega, Dübendorf, Switzerland). Measurements were performed on a HIDEX Sense microplate reader. Viability was calculated relative to the respective DMSO control (note that values higher than 100 % relative viability were not considered as biologically meaningful and were not followed up). Compounds inducing relative viability lower than 30 % were considered as active in this assay. IC_50_ calculations were carried out for ESI-09, testing this compound on metacestode vesicles at concentrations from 120 to 0.16 μM in 1:3 serial dilutions in triplicates. IC_50_ values were calculated in R using the 3 parameter Weibull type 1 for each of the three independent experiments (Ritler et al., 2020).

#### 2.4.3 Isolation of *E. multilocularis* germinal layer cells

The isolation of GL cells was conducted according to (Kaethner et al., 2023). Briefly, conditioned DMEM (cDMEM) was prepared prior to isolation. For the preparation of cDMEM, 1 × 10^6 RH cells and 10 × 10^6 RH cells incubated in DMEM supplemented with 10 % FBS, penicillin (100 U/mL), streptomycin (100 μg/mL) and tetracycline (5 μg/mL) for six or four days, respectively, at 37 °C under a humid, 5 % CO_2_ atmosphere. The supernatant of each was mixed 1:1 and sterile filtrated, resulting in cDMEM. Metacestode vesicles of at least 6 months of age were washed with PBS, incubated in distilled H_2_O for 2.5 min and subsequently mechanically disrupted. The resulting pellet was resuspended in 7 vol of pre-warmed 0.05 % Trypsin/0.02 % EDTA and incubated at 37 °C for 30 min. GL cells were extracted from the laminated layer by vigorous shaking followed by filtration through a 30 μm mesh (Sefar AG, Heiden, Switzerland) and separated from calcareous corpuscles by short centrifugation at 50×*g* for 30 s at RT. The isolated cells were centrifuged for 10 min at 600×*g* and RT and the pellet was resuspended in cDMEM. The cell suspension was diluted 1:100 in PBS to measure the OD_600_. An OD_600_ value of 0.1 of this dilution was defined as one arbitrary unit (AU) per μL of the undiluted cell suspension corresponding to 0.97 ± 0.11 μg of total protein for the GL cells as measured via Pierce™ bicinchoninic acid (BCA) Protein Assay Kit according to the manufacturer's protocol (Fisher Scientific AG, Reinach, Switzerland). 1′000 AU per 5 mL of cDMEM were cultured at 37 °C overnight under a humid nitrogen atmosphere. The next day, 2′000 AU of GL cells were combined and further cultured for 3 h at 37 °C under a humid nitrogen atmosphere before they were used for further experiments.

#### 2.4.4 Assessment of *E.* m*ultillocularis* germinal layer cell viability

To assess effects of compounds on the cell viability of isolated *E. multilocularis* GL cells, the protocol was applied as previously described (Kaethner et al., 2023). In short, 15 AU of GL cells were distributed into each well of a black 384-well plate. Compounds dissolved in DMSO (alexidine, carbendazim, ESI-09, MMV1581545 and oxfendazole) were added to final concentrations ranging from 80 to 0.31 μM (1:2 serial dilution) and the cells were cultured for 5 days at 37 °C under a humid, microaerobic atmosphere (85 % N_2_, 10 % CO_2_, 5 % O_2_). DMSO with the respective concentration was used as a solvent control. Viability was measured using the CellTiter-Glo® kit. Luminescence was measured using a HIDEX Sense microplate reader. Measurements were done in quadruplicates and represented as relative viability of the DMSO controls. IC_50_ values were calculated in R applying the five parameter logistic model for three independent experiments (Ritler et al., 2020).

### 2.5 Assessment of mammalian cell toxicity

Mammalian cell toxicity on RH cells was measured as described in Rufener et al. (2018) with few alterations. Briefly, confluent (50′000 cells/well) and pre-confluent (5′000 cells/well) were seeded into 96-well plates in DMEM supplemented with 10 % FBS, penicillin (100 U/mL), streptomycin (100 μg/mL) and tetracycline (5 μg/mL) and incubated at 37 °C under a humid, 5 % CO_2_ atmosphere for 24 h and 5 h respectively. Alexidine, ESI-09 and MMV1581545 were added to final concentrations ranging from 120 to 0.49 μM (1:3 serial dilution) to each well and then cells were incubated at 37 °C under a humid, 5 % CO_2_ atmosphere for 5 days. To measure viability, the medium was removed, 180 μL of PBS was added to the cells and resazurin was added to each well to a final concentration of 10 mg/L. The cells were then allowed to process the resazurin at RT for 1 h. Fluorescence was measured on a HIDEX Sense microplate reader. Measurements were performed in triplicates and IC_50_ values were calculated in R using the three parameter Weibull type 1 model for three independent experiments (Ritler et al., 2020).

### 2.6 Assessment of mitochondrial respiration in *E. multilocularis* germinal layer cells

To measure mitochondrial respiration in *E. multilocularis* GL cells, Seahorse “mito cell stress test” was performed according to the manual for Seahorse XFp Extracellular Flux Analyzer (Agilent Technologies, Basel, Switzerland) with some adaptations (Gu et al., 2021; Rufener et al., 2018). Seahorse XFp 8-well plates were hydrated and incubated together with Seahorse XF calibrant solution at 37 °C in a non-CO_2_ incubator overnight the day before the actual experiment. Seahorse XFp cell culture miniplates were coated with Cell-tak cell and tissue adhesive (Fisher Scientific, Schwerte, Germany) at 22.4 μg/mL 100 AU of GL cells were seeded per well in Seahorse DMEM supplemented with 1 mM pyruvate, 2 mM glutamine, and 10 mM glucose. Oligomycin was used at a final concentration of 1.5 μM. FCCP at 0.5 μM and ESI-09 at 2 μM and the corresponding DMSO concentration was used as a negative solvent control. Antimycin and rotenone were not used, as no effects were observed in *E. multilocularis* GL cells (S1 Fig). FCCP and ESI-09 were titrated (from 4 to 0.125 μM for ESI-09 and from 1.6 to 0.05 μM for FCCP) to find the maximum uncoupling concentration (S2 Fig). The experiment was repeated three times independently with two technical replicates. Relative OCR increase was calculated by subtracting the OCR before the addition of FCCP, ESI-09 or DMSO from the OCR after the addition, and dividing it by the OCR before addition.

### 2.7 TMRE assay for the assessment of the mitochondrial membrane potential

Tetramethylrhodamine ethyl ester (TMRE) assay was employed to assess effects on the mitochondrial membrane potential (Scaduto and Grotyohann, 1999). 100 AU of *E. multilocularis* GL cells in 180 μL cDMEM were seeded per well into a round bottom 96-well plate. ESI-09, FCCP or the respective amount of the solvent control DMSO was added to the GL cells to a final concentration of 10 μM and cells were incubated at 37 °C under a humid, nitrogen atmosphere for 1 h. Next, TMRE was added to the GL cells to a final concentration of 0.1 μM and the cells were incubated under a humid, nitrogen atmosphere at 37 °C for 30 min in the dark. Finally, the cells were washed three times with PBS and transferred to a black 384-well plate for imaging. Imaging was done on a Nikon Eclipse Ti 2 Spinning Disk (Cicero) microscope. Fluorescence intensity in cell aggregates was analyzed using Fiji (ImageJ, Version 2.14.0). Images were first processed by applying an appropriate threshold to distinguish aggregates from the background, further the watershed function was then used to separate closely associated aggregates. Integrated density was extracted for each aggregate and normalized to its area.

### 2.8 Transmission electron microscopy of *E. multilocularis* metacestode vesicles treated with ESI-09

To investigate induction of ultrastructural changes in *E. multilocularis* metacestode vesicles treated with ESI-09, transmission electron microscopy (TEM) was performed. Metacestode vesicles were treated with either 1 μM ESI-09 or the solvent control DMSO with subsequent incubation for 24 h at 37 °C under a humid, 5 % CO_2_ atmosphere. Processing and imaging was done as previously described (Kaethner et al., 2023).

## 3 Results

### 3.1 Screening of the Pandemic Response Box led to the identification of three compounds with *in vitro* activity against *E. multilocularis*

400 compounds from the Pandemic Response Box were overview-screened *in vitro* on *E. multilocularis* metacestode vesicles at 10 μM by PGI release as well as metacestode vesicle viability assay (S3 Table). This screen was carried out in singlets and resulted in 37 double positive compounds after 12 days of drug-incubation (Fig. 1A). Relative PGI release was also measured after five days of drug incubation (S3 Table), where eleven compounds showed relative PGI release higher than 20 %. Eight of these compounds were also double positive hits after twelve days of drug incubation. Two compounds, MMV1582496 (4-(benzylamino)-5-chloro-2,6-difluorobenzene-1,3-dicarbonitrile) and MMV688991 (nitazoxanide) were active in the PGI assay after twelve days but did not reduce the metacestode vesicle viability and were thus excluded from further testing. One compound, MMV1580849, led to a relative PGI release of 24.5 % after 5 days, but did not induce a PGI release higher than 20 % nor decrease metacestode vesicle viability after twelve days and thus was also excluded.

**Fig. 1:**
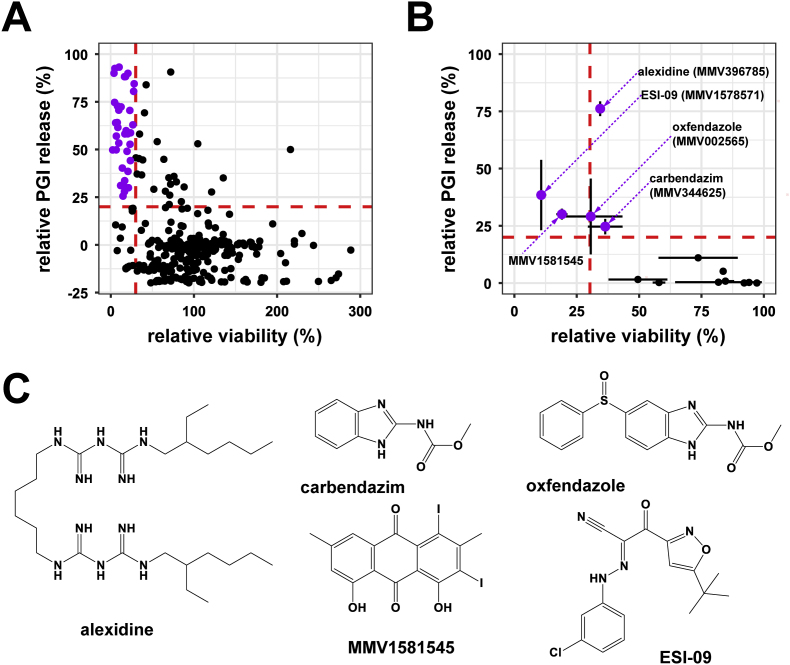
**Overview and confirmation screen of the Pandemic Response Box against*****E. multilocularis*****metacestode vesicles.** Compounds were tested at 10 μM and metacestode vesicles were incubated for 12 days at 37 °C under a humid, 5 % CO_2_ atmosphere. (A) Overview screen was done in singlets. Red dashed lines indicate cut-off for active compounds (PGI release ≥20 % and viability ≤30 %). Purple points represent active compounds. (B) Confirmation screen of the 37 initial hits. Red dashed lines indicate cut-off for active compounds (PGI release ≥20 % and Viability ≤30 %). Shown are means ± SD. (C) 2D structures of active compounds. Structures were generated using ChemDraw.

The initial 37 double positive compounds were subjected to a confirmation screen performed in triplicates. For four compounds activity was confirmed applying the same cut-off as for the overview screen (including the SD of the triplicates). Active compounds were carbendazim (MMV344625), ESI-09 (MMV1578571), MMV1581545, and oxfendazole (MMV002565) (Fig. 1B). As alexidine showed clear activity with a relative PGI release of 76.1 ± 3.2 % and a relative viability of 34.4 ± 0.9 % just slightly above the cut-off, alexidine was also included in further experiments. Respective molecule structures are shown in Fig. 1C.

To evaluate the parasiticidal potential of these five compounds, IC_50_ values were calculated for isolated *E. multilocularis* GL cells. Oxfendazole and carbendazim did not reduce GL cell viability at the tested concentration range and incubation time (S4 Fig), thus three compounds were identified with activity against *E. multilocularis*: alexidine, MMV1581545 and ESI-09. IC_50_ values on GL cells were calculated to be 10.73 ± 1.13 μM for alexidine, 7.89 ± 1.43 μM for MMV1581545, and 2.45 ± 0.86 μM for ESI-09 as seen Fig. 2 and Table 1 (all data is shown in S5 Fig).

**Fig. 2:**
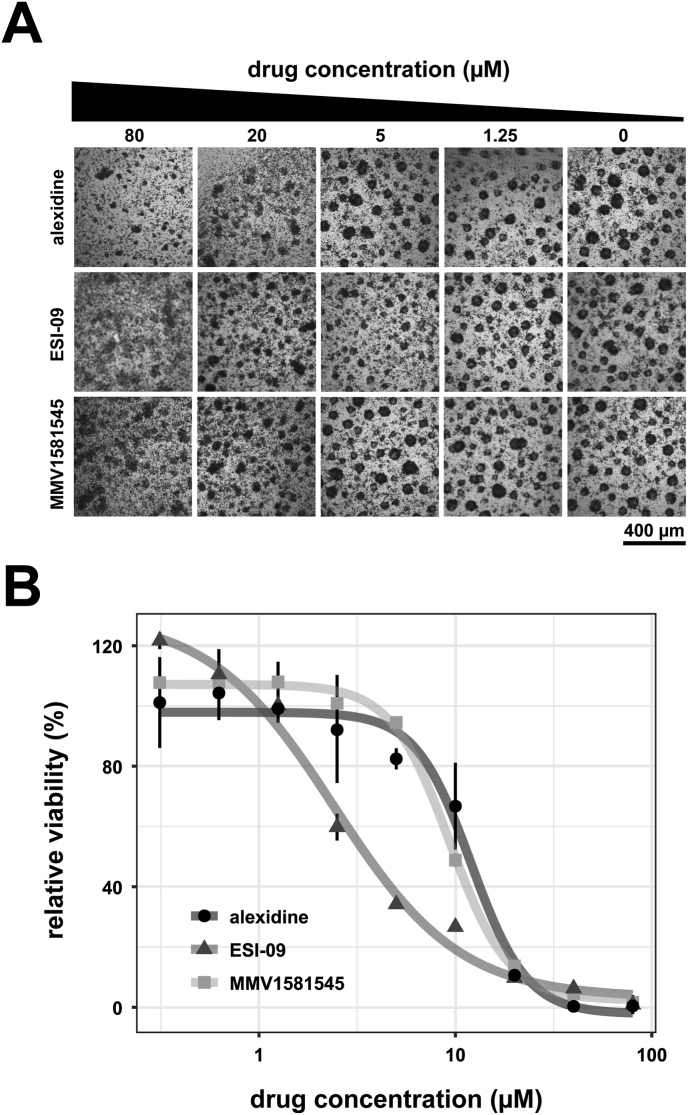
**Dose-response curves of the active compounds on GL cells.** Compounds (alexidine, ESI-09 and MMV1581545) were tested on isolated *E. multilocularis* GL cells at varying concentrations. Cells were incubated for 5 days under a microaerobic atmosphere at 37 °C. (A) Representative pictures of GL cell aggregates incubated with the different compounds and concentrations as indicated. (B) Viability of GL cells was tested using CellTiter-Glo® in quadruplicates and plotted relative to the solvent control DMSO. Shown are means ± SD and the non-linear regression models. Plot shows one representative experiment. The experiment was performed three times independently (S5 Fig).

**Table 1:** **Summarized parasite toxicity and mammalian cell toxicity of the five active compounds from the Pandemic Response Box as well as results from previous MMV library screenings (Malaria and Pathogen Box)**. IC_50_ values were calculated based on PGI release assay and metacestode viability assay (for *E. multilocularis* metacestode vesicles, readout after 12 days of incubation) and GL cell viability assay (for *E. mulitlocularis* GL cells, 5 days incubation), while resazurin assay was used for mammalian RH cells (5 days incubation). Means ± SD of three independent experiments are given. *P*-values for the therapeutic window were calculated using s two-sided *t*-test comparing the IC_50_ on pre-confluent RH cells to the IC_50_ on GL cells. Data taken from previous MMV library screenings is indicated by ∗ and ∗∗ for (Rufener et al., 2018; Stadelmann et al., 2016), respectively. ND: not defined.

	IC_50_ (μM)					therapeutic window
Compound	GL cells	Metacestode vesicles (viability assay)	Metacestode vesicles (PGI release assay)	confluent RH cells	pre- confluent RH cells	*p*-value
alexidine (MMV396785)	10.73 ± 1.13	ND	ND	5.97 ± 4.89	2.80 ± 1.33	0.23
ESI-09 (MMV1578571)	2.45 ± 0.86	2.09 ± 0.56	6.06 ± 3.18	37.33 ± 0.73	9.03 ± 1.30	7.09E-07
MMV1581545	7.89 ± 1.43	ND	ND	21.27 ± 4.39	14.65 ± 2.09	0.04
buparvaquone (MMV689480)∗	ND	ND	2.87 ± 1.45	17.42 ± 2.09	8.47 ± 3.89	ND
ELQ-400 (MMV671636)∗	ND	ND	0.02 ± 0.01∗	2.07 ± 1.67∗	0.25 ± 0.11∗	ND
MMV665807∗∗	0.6 ± 0.37∗∗	ND	1.2 ± 1.6∗∗	20.5 ± 0.13∗∗	4.80 ± 0.59∗∗	ND

### 3.2 ESI-09 exhibits specific toxicity against *E. multilocularis* germinal layer cells

The three compounds with parasiticidal activity were then counter screened on mammalian cells to assess specificity and the potential for a therapeutic window. All compounds were more toxic against pre-confluent cells compared to confluent cells. Alexidine was by far the most toxic compound with an IC_50_ of 5.97 ± 5.18 μM on confluent and 2.80 ± 1.34 μM on pre-confluent RH cells. MMV1581545 and ESI-09 were less toxic with IC_50_ values of 21.27 ± 4.39 μM and 37.33 ± 0.73 μM on confluent cells and 13.81 ± 1.09 μM and 9.02 ± 1.30 μM on pre-confluent cells, respectively (see Table 1 and S6 Fig). Given the high potency of ESI-09 against *E. multilocularis* GL cells and its significantly higher IC_50_ on pre-confluent RH cells (*p* = 7.09e-07), this compound was identified as the most potent with the broadest *in vitro* therapeutic window.

### 3.3 ESI-09 shows promising anti-echinococcal effects *in vitro*

To further examine the damaging and parasiticidal activity of ESI-09 on *E. multilocularis in vitro,* IC_50_ values using the PGI release and the metacestode vesicle viability assay were determined.

ESI-09 showed high efficacy against *E. multiloculari*s metacestodes after twelve days of incubation with calculated IC_50_ values of 6.06 ± 3.18 μM when measured by PGI release assay, and 2.09 ± 0.56 μM by metacestode viability assay, as seen in Fig. 3 and Table 1 (all experiments are shown in S7 Fig). Microscopy pictures clearly show the detachment of the GL from the laminated layer, visible from 13.3 μM on (Fig. 3A).

**Fig. 3:**
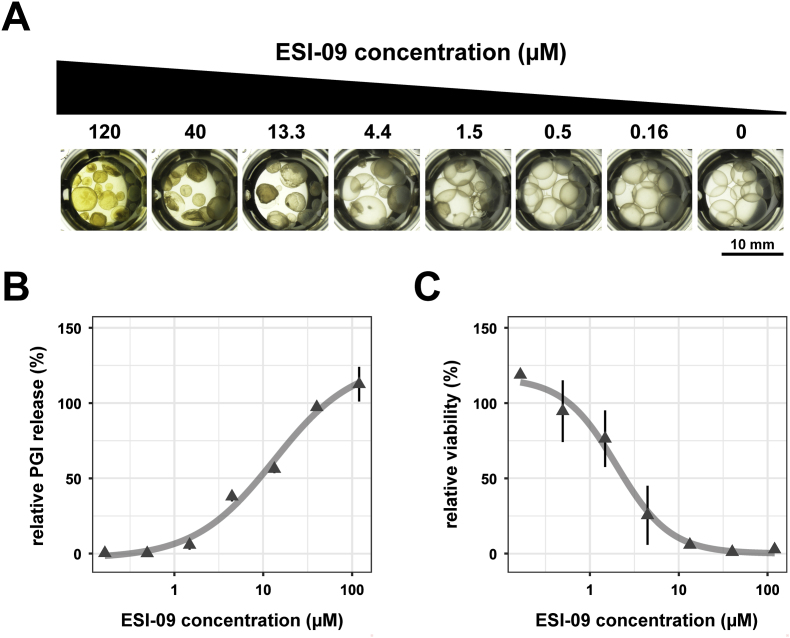
**Dose-response curves for ESI-09 tested on whole metacestode vesicles**. The effect of ESI-09 was tested at varying concentrations. Metacestode vesicles were incubated for 12 days under a humid, 5 % CO_2_ atmosphere at 37 °C and measurements were done in triplicates. Shown are means ± SD and the fitted non-linear regression model. Plot shows one representative experiment. This experiment was performed three times independently (S6 Fig.). (A) Representative pictures of metacestode vesicles treated with ESI-09 at different concentrations as indicated. Note the clear detachment of the GL from the laminated layer, which is visible from 13.3 μM on. (B) Damage-marker release relative to the total damage control Tx-100 measured after 12 days by PGI release assay. (C) Viability of metacestode vesicles relative to the solvent control DMSO measured after 12 days by metacestode vesicle viability assay.

### 3.4 ESI-09 acts on mitochondria in *E. multilocularis* germinal layer cells

As ESI-09 has been shown previously to act as a mitochondrial uncoupler in cancer cell lines (Maeda et al., 2020), we assessed the mitochondrial effects of this compound on *E. multilocularis* GL cells by Seahorse technology assays as well as TMRE assay. To establish the optimal uncoupling concentration, FCCP and ESI-09 were titrated, resulting in an optimal uncoupling concentration of 0.4 μM for FCCP and 2 μM for ESI-09 (S4 Fig). Upon addition of ESI-09 or FCCP to *E. multilocularis* GL cells, an increase of the oxygen consumption rate (OCR) was observed, as typically expected for a mitochondrial uncoupler (Fig. 4). On the other hand, the addition of the solvent control DMSO did not lead to an increase of the OCR. The relative increase of the OCR was significantly higher compared to DMSO for both ESI-09 and FCCP (*p* = 0.006). However, there was no significant difference between ESI-09 and FCCP, suggesting comparable uncoupling effects of both compounds, albeit at different drug concentrations (Fig. 4A). TMRE assays showed that the addition of ESI-09 led to a significant reduction of TMRE fluorescence in GL cell aggregates compared to the DMSO control (*p* = 6.42e-10). This reduction in fluorescence intensity was more pronounced in the FCCP-treated GL cells with a significant reduction to both DMSO (*p* = 8.97e-11) and ESI-09 (*p* = 5.01e-12) treated GL cells as seen in Fig. 4C. This experiment was repeated independently with similar results shown in S8 Fig.

**Fig. 4:**
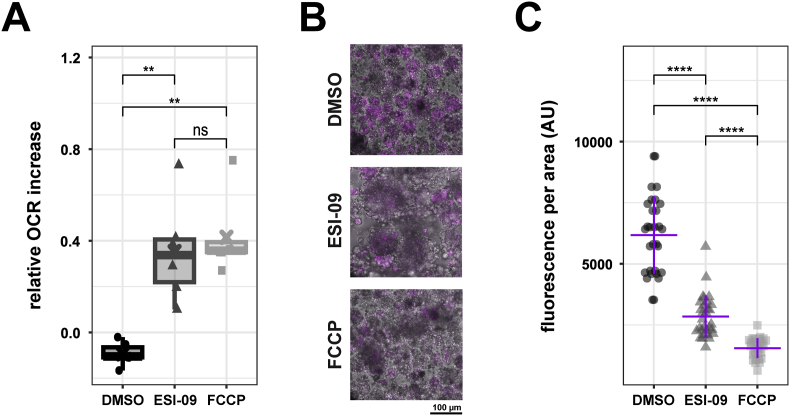
**ESI-09 acts as a mitochondrial uncoupler in*****E. multilocularis*****GL cells.** Oxygen consumption rate (OCR) was measured using Seahorse XFp analyzer in technical duplicates. (A) Relative OCR increase upon injection of either DMSO, 2 μM ESI-09 or 0.5 μM FCCP. Shown are individual data points of three independent experiments in the box plot. Pairwise comparisons were performed using the Wilcoxon Rank Sum Test with Bonferroni correction for multiple comparisons. Significance levels: 0.001 < p ≤ 0.01 (∗∗), p > 0.05 (ns). (B) TMRE fluorescence intensity was measured via confocal microscopy. GL cells were either treated with 10 μM ESI-09, FCCP or the solvent control DMSO. Representative microscopy pictures of maximal intensity z-stacks. (C) TMRE fluorescence intensity was measured via confocal microscopy. GL cells were either treated with 10 μM ESI-09, FCCP or the solvent control DMSO. Quantification of TMRE fluorescence signal normalized to GL cell aggregate area. Shown are single values of 30 individual aggregates. The purple crossbar represents the mean value, while the vertical bar represents the SD. Pairwise comparisons were performed using the Wilcoxon Rank Sum Test with Bonferroni correction for multiple comparisons. Significance levels: p ≤ 0.0001 (∗∗∗∗).

### 3.5 ESI-09 induces ultrastructural alterations in *E. multilocularis* metacestode vesicles

The ultrastructure of *E. multilocularis* metacestode vesicles was visualized by TEM. Non drug-treated metacestode vesicles exhibited the typical LL, tegument with microtriches and the GL tissue comprised of muscle cells, glycogen storage cells, as well as numerous mitochondria with clearly discernible cristae (Fig. 5A). Treatment with 1 μM ESI-09 for 24 h induced distinct ultrastructural changes (Fig. 5B–F). These included the formation of numerous electron-dense lipid droplets (Fig. 5C), phagosome-like structures (Fig. 5D) as well as a particularly high number of extracellular vesicle-like structures being released at the interface between the tegument and the laminated layer (Fig. 5E). These structures were not observed in the solvent control (Fig. 5A). However, no ultrastructural changes in mitochondria were seen. Fig. 5F, illustrates electron-dense mitochondria with cristae observed in ESI-09-treated metacestode vesicles.

**Fig. 5:**
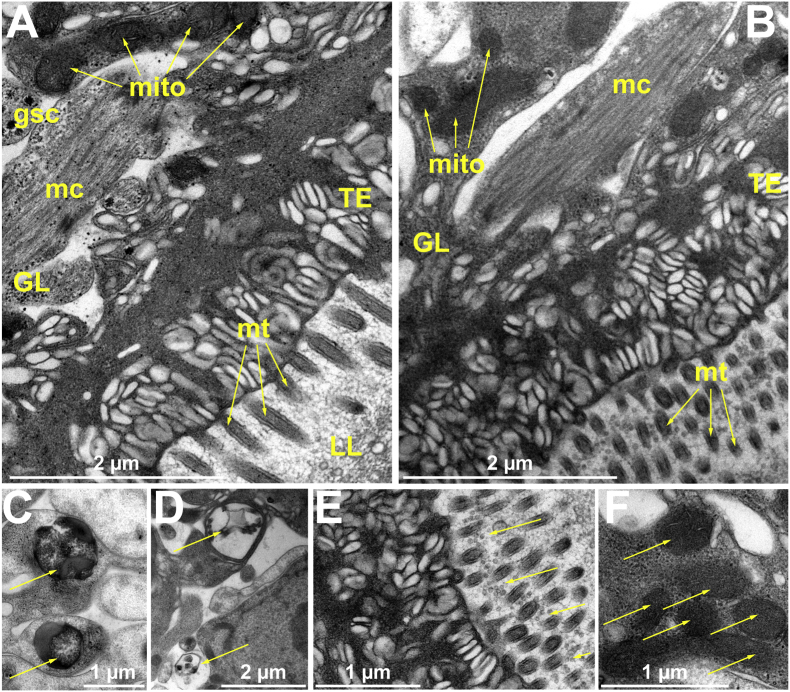
**Effects of ESI-09 on metacestode vesicle ultrastructure.***E. multilocularis* metacestode vesicles were treated either with 1 μM of ESI-09 or the solvent control DMSO for 24 h. Ultrastructural morphology of metacestode vesicles treated with (A) DMSO or (B) ESI-09. GL: germinal layer, LL: laminated layer, TE: tegument. gsc: glycogen storage cells, mc: muscle cell, mito: mitochondria, mt: microtriches. (C) Yellow arrows indicate electron dense lipid droplets observed after ESI-09 treatment. (D) Phagosome-like structures indicated by yellow arrows upon ESI-09 treatment. (E) ESI-09-induced accumulation of extracellular vesicle-like structures at the tegument-laminated layer interface indicated by yellow arrows. (F) Yellow arrows show electron-dense mitochondria with visible cristae upon ESI-09 treatment.

## 4 Discussion

AE is a life-threatening disease with low, but increasing, case numbers (Rostami et al., 2025). Today's drug treatment options are highly limited and are parasitostatic, inhibiting parasite growth rather than killing the parasite (Lundström-Stadelmann et al., 2019). Thus, new drug treatment options for AE are urgently needed. Establishment of *in vitro* culture techniques of *E. multilocularis*, including axenic cultures and GL cell cultures, as well as objective drug screening assays such as the PGI release assay or the metacestode vesicle and GL viability assays have paved the way for medium-throughput drug screening against *E. multilocularis* (Kaethner et al., 2023; Lundström-Stadelmann et al., 2024; Spiliotis et al., 2010; Spiliotis and Brehm, 2009; Stadelmann et al., 2010).

In this study, we screened the 400 compounds included in the open-access Pandemic Response Box against *E. multilocularis in vitro* by PGI release assay and metacestode vesicle viability assay. One limitation of our drug screening approach is the extended incubation period of 12 days, which was necessary due to the nature of the damage-marker PGI release assay employed. This assay, which relies on the accumulation of a parasite-derived damage-marker in culture supernatants, requires prolonged exposure to compounds to detect effects, especially for slow-acting drugs. However, this extended duration raises the possibility that some compounds may be metabolized, degraded, or lose potency over time, potentially underestimating their efficacy. Medium replacement was not feasible during the incubation period, as it would have removed the released damage-marker and compromised the assay's readout. To complement the damage-marker release assay, we implemented a secondary viability assay after 12 days of drug exposure to further validate compound activity. Interestingly, some discrepancies were observed between the two assays. For example, the drug nitazoxanide showed activity in the PGI release assay, but was excluded from further screening as it did not meet the threshold in the metacestode viability assay. Interestingly, anti-echinococcal activity of nitazoxanide was previously demonstrated against *E. multilocularis* as well as *E. granulosus in vitro*, again highlighting the importance of assay choice in a screening approach (Laura et al., 2015; Stettler et al., 2003).

Our screening of the open-access library revealed four active compounds, namely, carbendazim (MMV344625), ESI-09 (MMV1578571), MMV1581545 and oxfendazole (MMV002565), but also alexidine (MMV396785) (clear activity, however slightly above cut-off relative viability). These were then further evaluated against isolated GL cells. The GL cell population is made of about 25–30 % stem cells, which are the only mitotic cells in the parasite and give it its high regenerative ability (Brehm and Koziol, 2014). Thus, compounds that are highly effective against GL cells suggest parasiticidal activity and are therefore considered to be promising for future treatments. Carbendazim and oxfendazole were not able to decrease GL cell viability, whereas alexidine, ESI-09 and MMV1581545 were highly effective against them, with ESI-09 being the most potent. Inactivity of the BMZs (carbendazim and oxfendazole) against isolated GL cells was to be expected, as the stem cells in the GL cell population mainly express the *tub-2* isoform of beta-tubulin, containing amino acid motifs that are linked to BMZ resistance (Schubert et al., 2014). Carbendazim is a systemic fungicide extensively used in agriculture and forestry to combat plant fungal infections. Additionally, it serves as a fruit preservative and is utilized in industries such as paint, textiles, and papermaking. Despite its wide range of applications, carbendazim has been linked to environmental contamination and harmful effects on non-target organisms and thus the World Health Organization (WHO) has classified it as a hazardous substance, prompting bans in Australia, the United States, and many European Union countries (Singh et al., 2016). Oxfendazole on the other hand as a broad-spectrum anthelminthic applied in veterinary medicine to control and treat infections caused by gastrointestinal cestodes and nematodes in various animal species (Gonzalez et al., 2019). Interestingly, oxfendazole is metabolized into fenbendazole, which itself has anthelminthic activity (Gonzalez et al., 2019). Efficacy of oxfendazole was demonstrated against cystic echinococcosis in ruminants as well as porcine cysticercosis caused by the related cestode *Taenia solium* (Gavidia et al., 2010; Nsadha et al., 2021). Activity of oxfendazole's metabolite fenbendazole against *E. multilocularis* was shown *in vitro* as well as *in vivo* with effects comparable to albendazole (Küster et al., 2014)*.* Fenbendazole is included in the Pandemic Response Box, but was excluded during the overview screen, which is in alignment with its BMZ typical inactivity in the PGI release assay *in vitro* when incubated for short time-intervals (Küster et al., 2014).

To assess whether the toxicity of alexidine, ESI-09 and MMV1581545 was parasite-specific, they were counter-screened against a mammalian cell line. MMV1581545 and ESI-09 only showed moderate toxicity, while alexidine was highly toxic. Alexidine's toxicity is in line with previously published results, where it induced severe toxicity in the HUVEC cell line at 15 μM (Mamouei et al., 2018). The bis-biguanide compound alexidine is related to chlorhexidine and was initially described as a disinfectant with antibacterial properties, which lead to its application as an anti-plaque mouthwash (Barrios et al., 2013; Mamouei et al., 2018). The elevated mammalian cell toxicity also led to the exclusion of alexidine from screens in other organisms and supports the current practice of limiting alexidine only to topical application (Biendl et al., 2022; Nabeela et al., 2022). MMV1581545 is a halogenated derivative of emodin, which exhibits antibacterial activity (Duan et al., 2014). Furthermore, it was shown that emodin has antiparasitic properties with activity against various organisms including *Toxoplasma gondii*, *Trypanosoma brucei* and *Plasmodium falciparum* (Semwal et al., 2021).

Thus, due to the toxicity of alexidine and the limited data available for MMV1581545 and its lower efficacy compared to ESI-09, we further focused on the compound ESI-09. ESI-09 was tested again to measure the IC_50_ on whole metacestode vesicles, as compounds can be very effective on GL cells, but not on whole metacestode vesicles. Also on whole metacestode vesicles, ESI-09 was efficacious. Compared to lead compounds obtained in previous MMV library screens (Table 1) against *E. multilocularis*, ESI-09 appeared to be less potent, with an IC_50_ of 6.06 ± 3.18 μM on whole metacestode vesicles and 2.45 ± 0.86 μM on isolated GL cells after twelve days of incubation. For example, the lead compound of the Malaria Box, MMV665807, exhibited an IC_50_ of 1.2 ± 1.6 μM on whole metacestode vesicles and 0.6 ± 0.37 μM on GL cells, albeit with higher mammalian toxicity, as indicated by an IC_50_ of 20.5 ± 0.13 μM on confluent RH cells an IC_50_ of 4.80 ± 0.59 μM on pre-confluent RH cells versus 37.33 ± 0.73 μM and 9.03 ± 1.30, respectively, for ESI-09 (Stadelmann et al., 2016). Similar findings were reported from the Pathogen Box, where buparvaquone exhibited an IC_50_ of 2.87 ± 1.45 μM on metacestode vesicles and 17.42 ± 2.09 μM and 8.47 ± 3.89 μM on confluent and pre-confluent RH cells, respectively. ELQ-400 exhibited an IC_50_ of 0.02 ± 0.01 μM on metacestode vesicles and IC_50_ values of 2.07 ± 1.67 μM on confluent RH cells and 0.25 ± 0.11 μM on pre-confluent RH cells (Rufener et al., 2018). Of note is, that in these previous studies, the IC_50_ values on metacestode vesicles were measured by PGI release after five days and in the present study we calculated the IC_50_ of ESI-09 by PGI release after twelve days of drug incubation, potentially further decreasing the IC_50_ for ESI-09 in comparison to the mentioned compounds. In summary, ESI-09 exhibited promising parasiticidal effects with low enough mammalian toxicity to provide a potential therapeutic window.

The non-cyclic nucleotide antagonist ESI-09 was first discovered to be an exchange protein activated by cAMP (EPAC) inhibitor through a high-throughput screening using purified human EPAC enzymes (Almahariq et al., 2013). As the name suggests, EPACs are sensors of the secondary messenger cAMP. Activated by cAMP they interact with Ras-like GTPases and act as a guanine nucleotide exchange factor (Roscioni et al., 2008). Involved in these signaling pathways, EPACs are important regulators of processes including calcium handling, cell proliferation, cell survival, cell differentiation, cell polarization, cell–cell adhesion events, gene transcription, secretion, ion transport, and neuronal signaling (Roscioni et al., 2008). ESI-09 acts as a competitive inhibitor competing with cAMP for the cAMP binding site (Almahariq et al., 2013; Zhu et al., 2015). Since then ESI-09 was shown several times to be effective against cancer cells *in vitro* (Almahariq et al., 2013; Kumar et al., 2017; Wang et al., 2017)*.* Furthermore, ESI-09 was shown to protect mice from bacterial rickettsioses and hindered replication of Middle East respiratory syndrome coronavirus *in vitro* (Gong et al., 2013; Tao et al., 2014). There is a putative EPAC homologue (EmuJ_00152650) in the genome of *E. multilocularis*, however, whether it is functional and how and is a target of ESI-09 has yet to be elucidated. The presence of and functionality of EPAC-interacting proteins in *E. multilocularis* may suggest functional involvement of the putative EPAC homologue (Spiliotis and Brehm, 2004).

More recently, ESI-09 was described as a mitochondrial uncoupler in cancer cell lines (Maeda et al., 2020). To assess whether ESI-09 acts on the mitochondria in *E. multilocularis*, we employed different techniques including Seahorse technology assays, TMRE assays and TEM. Using Seahorse technology assay we proved an uncoupling effect of ESI-09 in *E. multilocularis* GL cells, providing evidence for the inner mitochondrial membrane as a potential target of ESI-09 in this parasite. We observed maximal uncoupling effects of ESI-09 at 2 μM, which was comparable to the maximal uncoupling activity of FCCP, albeit at a higher concentration. This is consistent with the previously reported optimal uncoupling concentration of ESI-09 of 2 μM observed in cancer cell lines (Maeda et al., 2020). Furthermore, we observed decreased TMRE fluorescence when *E. multilocularis* GL cells were treated with ESI-09, indicating ESI-09 affecting the mitochondrial membrane potential. This effect was less compared to FCCP again indicative of its lower uncoupling potency. TEM analysis on treated metacestode vesicles was performed to assess morphological changes at the cellular level, particularly focusing on the parasite's mitochondria. However, the observed uncoupling of ESI-09 did not significantly affect the morphology of mitochondria. Phagosome formation, increased release of extracellular vesicle-like structures into the laminated layer, and potential aggregates in lipid droplets, were noted. Mitochondrial uncoupling is known to induce mitophagy (Demine et al., 2019), which could be attributed to the phagosome-like structures observed after ESI-09 treatment of metacestode vesicles. Concluding, our experiments suggest the inner mitochondrial membrane of the parasite as one of the targets of ESI-09. Interestingly, many compounds that were described to be active against *E. multilocularis* also affect the mitochondria, e.g. the complex III inhibitors ELQs and buparvaquone identified in the MMV Pathogen Box screening before (Chaudhry et al., 2022; Rufener et al., 2018), or ascofuranone, a complex II/III inhibitor (Enkai et al., 2023).

Whether ESI-09 shows efficacy against AE *in vivo* is yet to be determined. Recent *in vivo* studies in mice have demonstrated ESI-09's activity in ameliorating pathological conditions (Ahmed et al., 2019; Zhu et al., 2015). Daily dosages of 10 mg/kg via intraperitoneal treatment for 21 days or 50 mg/kg via oral gavage up to 26 days have proven to be well-tolerated in mice (Almahariq et al., 2013; Maeda et al., 2020). However, to date there is no study measuring pharmacokinetics and drug serum levels. Thus, it is questionable if the available data for this compound would allow future application as salvage treatment for human AE patients.

In conclusion, we have identified ESI-09 as an effective compound against *E. multilocularis* metacestode vesicles *in vitro* by screening the open-access Pandemic Response Box. ESI-09 showed high parasiticidal effects *in vitro* with moderate mammalian cell toxicity. Furthermore, we provide evidence for the inner mitochondrial membrane as one of the targets of ESI-09 in *E. multilocularis*, which also sheds light on a potentially exploitable metabolic vulnerability in *E. multilocularis.*

## CRediT authorship contribution statement

**Pascal Zumstein:** Writing – review & editing, Writing – original draft, Visualization, Methodology, Investigation, Formal analysis, Data curation, Conceptualization. **Anissa Bartetzko:** Writing – review & editing, Visualization, Methodology, Investigation, Formal analysis, Data curation, Conceptualization. **Marc Kaethner:** Writing – review & editing, Supervision, Methodology. **Laura Vetter:** Writing – review & editing, Investigation. **Andrew Hemphill:** Writing – review & editing, Visualization, Methodology, Investigation, Data curation. **Trix Zumkehr:** Writing – review & editing, Investigation. **Benoît Laleu:** Writing – review & editing, Resources. **Matías Preza:** Writing – review & editing, Writing – original draft, Supervision, Methodology, Investigation, Formal analysis, Conceptualization. **Britta Lundström-Stadelmann:** Writing – review & editing, Writing – original draft, Visualization, Supervision, Resources, Project administration, Funding acquisition, Formal analysis, Conceptualization.

## Funding statement

This study was supported by grants to 10.13039/100012059BLS from the 10.13039/501100017659Uniscientia Foundation and the Gottfried and Julia Bangerter-Rhyner Foundation, as well as the Swiss National Science Foundation (SNSF) grant number 310030_192072 and 320030_236056.

The funders had no role in study design, data collection, analysis and interpretation, decision to publish, or preparation of the manuscript.

## Conflict of interest declaration

I hereby confirm in the name of all listed authors (see below) for the manuscript „*In vitro* screening of the open-access Pandemic Response Box reveals ESI-09 as a compound with activity against *Echinococcus multilocularis*” that **NONE** of the authors have any conflict of interest. Thus, declarations of interest: none.

## Figures and Tables

**figs1:**
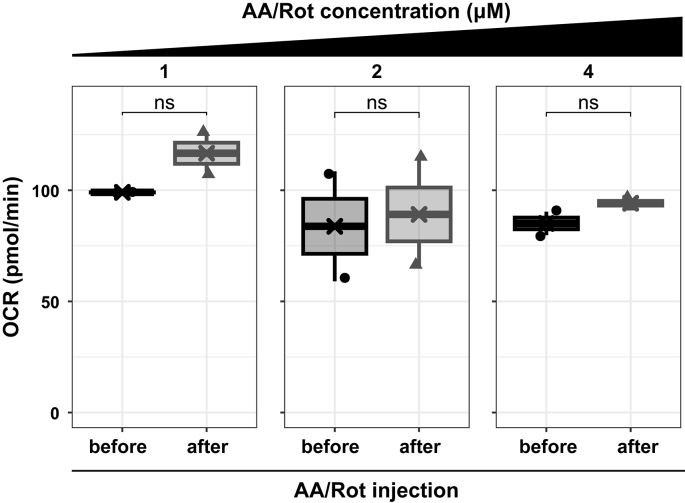


**figs2:**
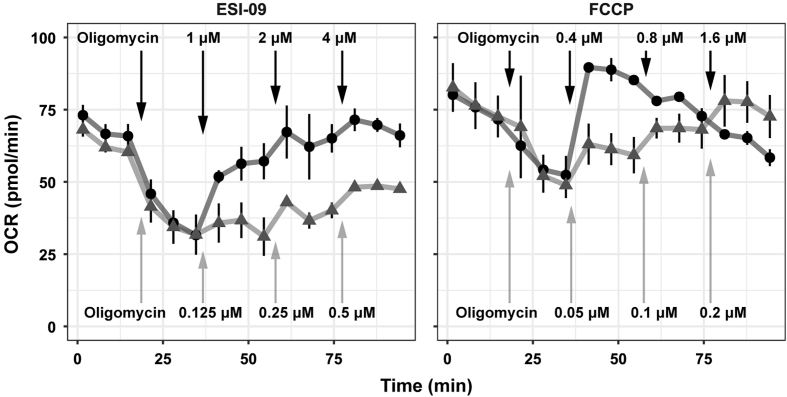


**figs3:**
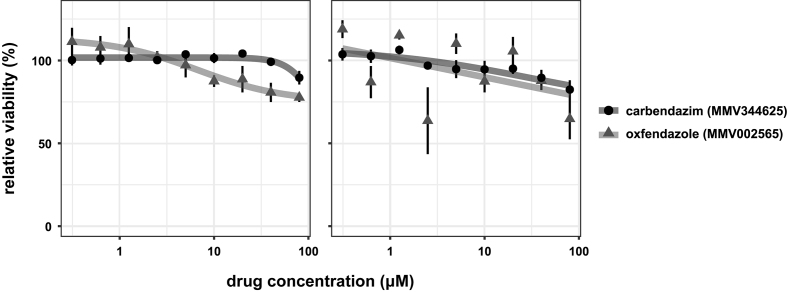


**figs4:**
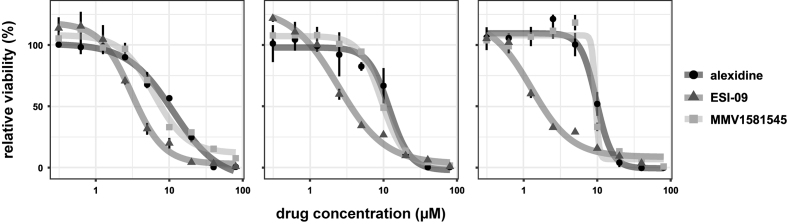


**figs5:**
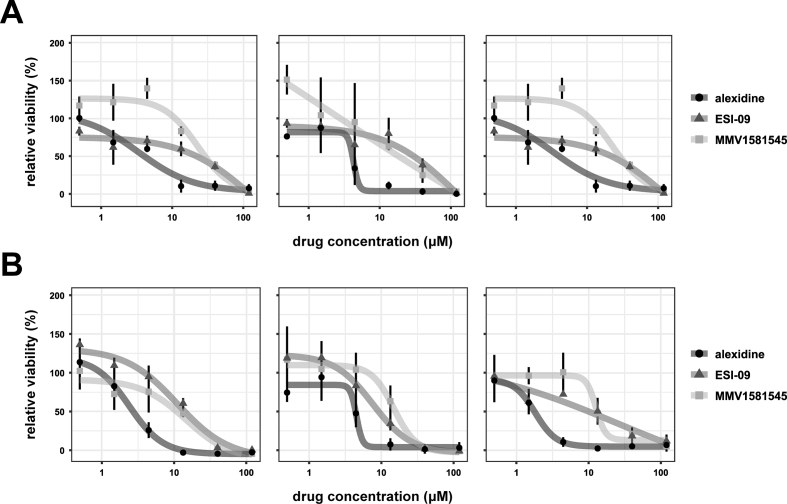


**figs6:**
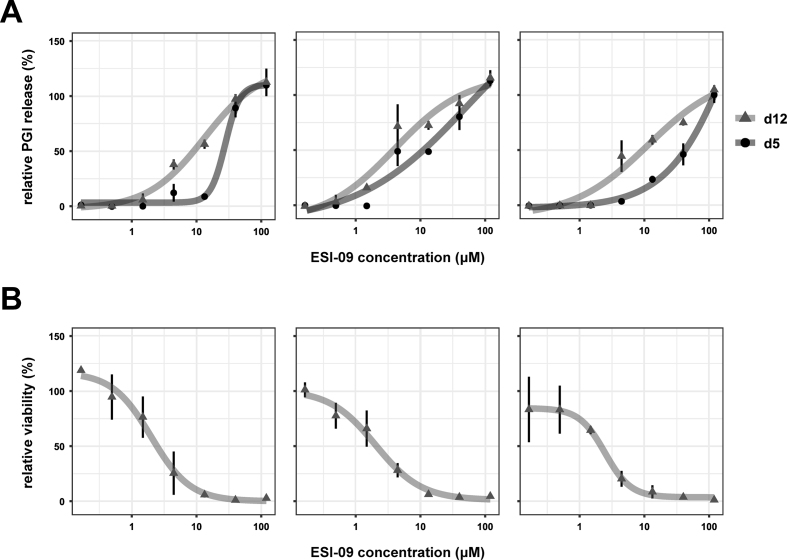


**figs7:**